# A Beginner’s Guide to Applying Large Language Models in Behavioral Interventions

**DOI:** 10.2196/79302

**Published:** 2026-04-29

**Authors:** Nirali Shah, Lorraine Buis, Derek Papierski, Alexis Castellanos, Marvin Mlakha, Susan Murphy

**Affiliations:** 1 Department of Physical Medicine and Rehabilitation University of Michigan Ann Arbor, MI United States

**Keywords:** behavior, digital health, intervention, large language model, LLM, behavioral intervention, self-management

## Abstract

Digital behavioral interventions are increasingly used to support chronic disease self-management, yet many systems rely on predetermined content that limits personalization and sustained engagement. Large language models (LLMs) offer new opportunities to deliver conversational behavioral support. However, integrating LLMs into behavioral interventions requires careful architectural, methodological, and ethical planning, which may be challenging for researchers without formal training in artificial intelligence. This viewpoint provides a structured introduction to LLMs tailored to behavioral science. We describe foundational concepts in natural language processing and transformer-based architectures, outline the core components of LLM-based systems, including prompting strategies, context management, retrieval-augmented generation, and guardrails, and illustrate these principles through our experience integrating a proprietary LLM into a mobile self-management intervention for individuals with systemic sclerosis. Building on this case example, we propose a phased design workflow to guide early-stage development and responsible implementation, along with a decision framework to help researchers navigate scientific and logistical trade-offs between proprietary models and other alternatives. The considerations presented here are informed by formative implementation efforts and are intended to support early-stage design decisions for LLM-based behavioral interventions. As these interventions continue to evolve, rigorous evaluation and interdisciplinary collaboration will be important to ensure that these systems improve personalization and scalability while maintaining safety and scientific rigor.

## Introduction

Behavioral interventions are treatment approaches designed to help individuals change behavior and develop skills to manage their chronic health conditions [1]. These interventions typically involve an interactive process between the individual and a health care provider and draw on evidence-based behavior change strategies, such as education, identification of patterns, motivation, goal setting, self-monitoring, evaluation, and reinforcement [1,2]. Despite evidence supporting their effectiveness in improving health outcomes [3-5], behavioral interventions are inconsistently implemented in primary care settings due to a shortage of trained providers, limited access, implementation costs, and insurance coverage constraints [6-8]. These challenges hinder scalability, personalization, and sustained engagement, thereby limiting the broader impact of behavioral interventions [9].

While digital technologies, including SMS text messaging, mobile health (mHealth), and web-based platforms, have been increasingly adopted for disease management [10,11], many systems rely on predetermined content or predefined if-then rules that do not adapt to changes in user behavior, engagement patterns, or contextual factors over time [12-14]. Digital interventions with limited tailoring or personalization are associated with lower engagement and ultimately reduced effectiveness in supporting sustained behavior change and disease management [12,15]. These limitations underscore the need for digital behavioral interventions that are both scalable and responsive to individual needs as they evolve.

## Natural Language Processing and Large Language Models

Artificial Intelligence (AI) refers to technologies that simulate human intelligence, including learning, reasoning, and decision-making [16]. Within this landscape, natural language processing (NLP) plays a central role in enabling machines to analyze and generate human language [17]. Historically, NLP relied on rule-based systems that used predefined grammars and pattern matching to classify text [17,18]. While effective for well-defined tasks, these systems struggled with the nuance of human conversations and were limited in their ability to generalize across different conversational contexts. For example, in the statement, “I’m exhausted, so I didn’t go for a walk today,” a rule-based system might flag the keywords “didn’t go” and simply classify the statement as an instance of nonadherence. Subsequent statistical models, such as recurrent neural networks, improved on this by processing text sequentially, one word at a time [19]. However, these models often suffered from the “vanishing gradient” problem, where the mathematical influence of earlier words weakened by the time the model reached the end of a long or complex sentence [20]. In the example statement “I’m exhausted, so I didn’t go for a walk today,” the recurrent neural network model might lose the semantic link between “exhausted” and “didn’t go,” resulting in a fragmented analysis that overlooks the underlying cause of the missed physical activity. Within the context of a behavioral intervention, understanding the underlying factors of nonadherence, such as low motivation, emotional burnout, lifestyle demands, or symptoms (eg, pain or fatigue), is crucial for delivering appropriate behavioral support, yet such nuance is difficult to capture using fixed rules or narrowly trained models.

Recent advances in NLP center on transformer architectures, which use self-attention mechanisms to process all words in a sequence simultaneously, allowing the model to weigh the relevance of each word relative to every other word in the message, thus preserving the causal and contextual information central to behavioral interventions [21]. Thus, in the example stated earlier, “I’m exhausted, so I didn’t go for a walk today,” the transformer-based model is able to capture the relationship between “exhausted” and “didn’t go for a walk,” preserving the understanding that the individual did not go for a walk because they were exhausted. Training transformer architecture models on massive datasets has given rise to large language models (LLMs) with emergent capabilities [22]. A defining feature of LLMs is “in-context learning,” which allows the model to adapt its interpretation and performance via specific instructions or examples through a technique called “prompting.” This includes “zero-shot prompting” (following instructions without examples) and “few-shot prompting” (guided by a small number of task-relevant examples) [22]. This flexibility allows LLMs to be adapted to specific clinical contexts through instruction rather than requiring the model to be built or retrained from scratch. While this can be highly beneficial in behavioral health settings, applying LLMs to interventions requires careful design and oversight because model outputs are generated probabilistically and may occasionally produce inaccurate, misleading, or clinically inappropriate responses if not properly constrained. Therefore, responsible integration of AI systems, including the use of grounding strategies, guardrails, and systematic evaluation, is essential when using LLMs for health-related interventions.

Although LLMs are increasingly adopted in behavioral and nonpharmacological treatments [23], few resources provide practical guidance on the foundational concepts and their responsible integration into interventions. This viewpoint paper aims to bridge this gap by providing a perspective-driven, practice-informed, structured introduction to core concepts, informed by our firsthand experience integrating LLMs into an mHealth intervention for individuals with systemic sclerosis (SSc) called resilience-building energy management to enhance well-being (RENEW) [24].

## RENEW Intervention

The RENEW intervention is a 12-week mHealth, peer-supported fatigue self-management intervention developed for individuals with SSc [24]. The original intervention was grounded in a positive psychology framework and paired participants with trained peer health coaches who provided individualized guidance to support behavior change across domains, such as physical activity, activity pacing, relaxation techniques, adaptive thinking, self-care, nutrition, and sleep, through structured, real-time video-based coaching sessions [24]. The health coaches were trained in motivational interviewing and followed a manualized protocol with ongoing supervision to support intervention fidelity. Although the original RENEW intervention demonstrated clinical benefit [24], sustaining a peer-delivered coaching model presented challenges related to personalization, scalability, cost, and long-term availability of trained coaches. These constraints motivated the integration of LLM-based systems into the RENEW platform through the RENEW-AI and RENEW via Artificial Language (RENEWAL) projects.

## RENEW-AI and RENEWAL Projects

In this paper, we describe key lessons learned from integrating an LLM into the RENEW intervention and iteratively refining it to deliver behavioral coaching content aligned with our program goals. This work was carried out in 2 phases: the RENEW-AI and RENEWAL projects. In RENEW-AI, we incorporated a chat-based interface within the RENEW mobile app that used a proprietary LLM (ie, GPT-4o; OpenAI) to provide disease-related education and behavioral health coaching consistent with the principles of the original peer-led RENEW program. In this initial iteration, responses of the AI health coach were shaped through text-based prompts such that the chat-based health coach reflected the tone, scope, and boundaries of peer health coaching provided in the original RENEW intervention. We emphasized concepts of motivational interviewing, empathy, encouragement, goal reflection, and self-management strategies, while explicitly excluding medical advice, diagnosis, or treatment recommendations.

Building on this foundation, the RENEWAL project extends the system architecture with a curated, evidence-based knowledge base of disease-specific materials. This approach allowed us to ground the model’s responses in program-specific content, ensuring that conversational outputs were clinically accurate. In addition to providing standardized education, the RENEWAL framework delivers personalized behavioral health coaching tailored to individuals’ reported symptoms. Specifically, the system adapts behavioral goals based on the participants’ reported symptoms at baseline. To ensure safety, the system is governed by guardrails that prevent the LLM from engaging in clinical decision-making or medical diagnosis.

## Overview of LLM Systems

**Figure 1:**
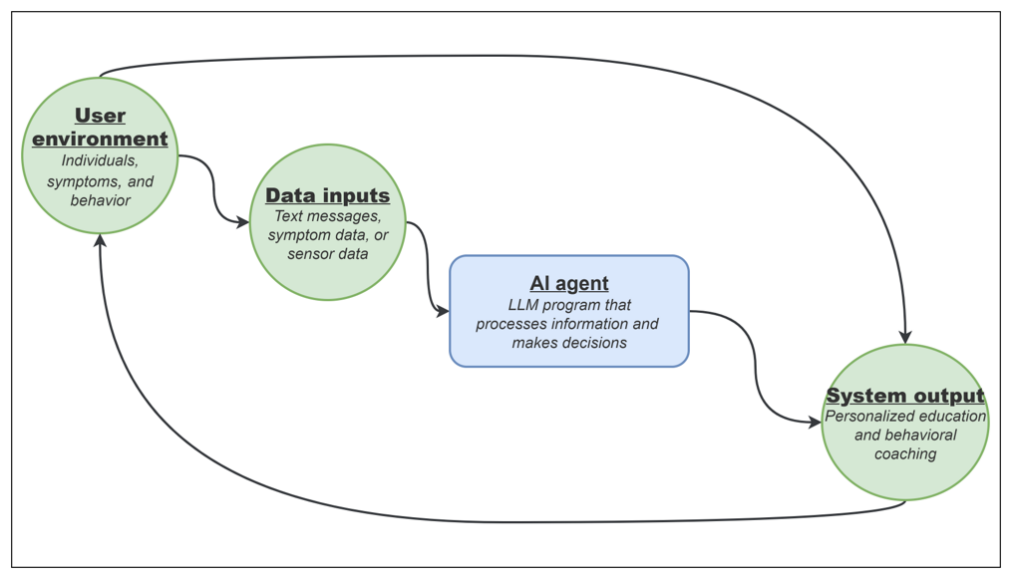
Overview of a large language model (LLM)–based behavioral intervention. This closed loop illustrates the dynamic interaction between the user, data inputs, the artificial intelligence (AI) agent, its underlying LLM, and system outputs.

An LLM-based behavioral intervention typically consists of a user environment, data inputs, the AI agent, and system outputs (Figure 1).

### User Environment

The user environment refers to the real-world context in which an individual interacts with the LLM system [25]. The environment includes contextual factors (eg, time of day, social setting, and symptoms) and behavioral factors (eg, physical activity, daily routines, and medication adherence) that influence both health status and device use patterns. In the RENEW-AI and RENEWAL projects, the user environment includes individuals living with SSc and their lived experience of the disease, including fluctuating symptoms and behaviors, which influence their interaction patterns with the AI health coach.

***Data Inputs***

Data inputs refer to the information collected from the user environment that the LLM system processes to generate system outputs [25]. The inputs may be structured (eg, survey responses) or unstructured (eg, text messages). In cases where passive data streams (eg, wearable sensor data) are incorporated, a preprocessing or translation layer is required for the LLMs to convert raw time-series data into structured features or text-based summaries that can be interpreted by the model as conversational context.

In RENEW-AI, primary data inputs consist of text messages entered by individuals with SSc through the chat functionality of the RENEW mobile app. In RENEWAL, additional input includes user-reported symptoms collected using survey data, which the system uses to provide disease-specific education and personalized health coaching.

### AI Agent

An AI agent is an interactive system that receives data input, processes information, and generates output in response to user interactions [25].

In both RENEW-AI and RENEWAL, the LLM agent is a conversational health coach integrated into the RENEW mobile app. The function of the agent is determined by its underlying LLM program, components of which are described in the following subsection.

### System Output

System output refers to the responses generated by the LLM agent and delivered to the user [25]. In LLM-based systems, system outputs are typically conversational text messages.

In both RENEW-AI and RENEWAL, system outputs include health coaching text responses designed to support reflection, reinforce self-efficacy, and guide self-management behaviors. The system outputs of the LLM agent influence the user environment by shaping users’ perceptions, motivation, and actions, thereby completing a feedback loop in which subsequent interactions generate new data inputs for the system.

## LLM Agent and Programs

### Overview

The operational architecture of an LLM-based behavioral intervention is driven by the LLM agent, the system’s decision-making logic, and its underlying program, which provides the computational framework to generate natural language. While the agent serves as the system’s “reasoning” layer, the underlying LLM program generates language by estimating the probability of the next unit of text (token) based on the preceding user context [21,26].

**Figure 2:**
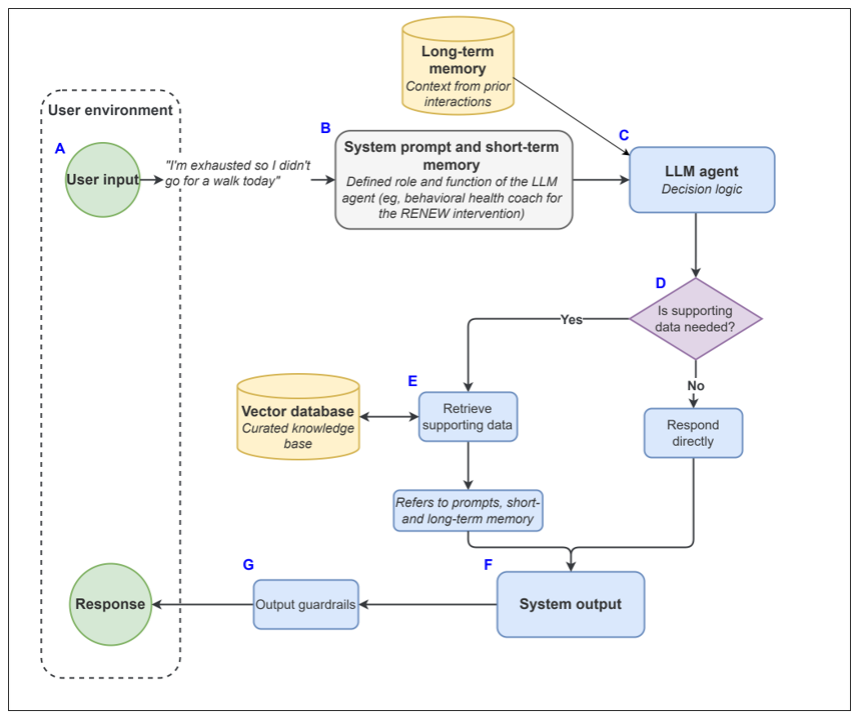
Architectural workflow of the large language model–based behavioral intervention. Labeled components (A-F) correspond to key stages in the system, including (A) user input, (B) memory integration, (C) large language model (LLM) agent, (D) decision-making, (E) retrieval-augmented generation, (F) system output, and (G) output guardrails. RENEW: Resilience-Building Energy Management to Enhance Well-being.

To describe the role of LLM agents and their underlying programs, we present the architectural configuration adopted in the RENEW-AI and RENEWAL projects. This configuration serves as a reproducible template for the design and implementation of future LLM-based behavioral interventions. As shown in Figure 2, the system workflow progresses through a series of components, from user input (A) to system output and guardrails (F-G).

### User Input

This process is initiated within the user environment when a user provides input (eg, “I’m exhausted so I didn’t go for a walk today”). This input serves as the primary data source for ongoing processing by the LLM system.

### Prompting and Memory Integration

The user input is combined with system-level instructions and contextual information*.*

*System-level instructions* are given through a process called “prompting.” Prompting refers to the specification of instructions, roles, tone, boundaries, and output expectations provided to an LLM [27,28]*.* Prompting can be done either using instructions alone (zero-shot prompting) or using a combination of instructions and examples of input-output pairs (few-shot prompting). This approach allows the model to respond in a consistent manner without modifying the model’s underlying parameters [22]. For behavioral interventions, prompt design may include the LLM’s role (eg, behavioral health coach using motivational interviewing principles), interaction style (eg, empathic and reflective), and explicit boundaries (eg, avoiding medical advice or diagnosis). In the RENEW-AI and RENEWAL projects, the system prompts defined the model as a supportive peer health coach that used motivational interviewing techniques and reflective listening. The prompts also included explicit instructions to avoid making medical diagnosis or treatment recommendations.

*Contextual information* is provided to the system using short-term memory (current conversation history) and long-term memory (user summaries from prior interactions). The context window in an LLM system refers to the model’s working memory, that is, the total volume of information the model can consider at a given time during a conversation. This typically includes data inputs, prompt instructions, context from the current conversation, and context from accumulated conversation history. Information that exceeds the context window is no longer available to the model’s immediate processing unless it is explicitly reintroduced or stored through system-level mechanisms. For behavioral interventions, the standard context window (short-term memory) may be insufficient. Consequently, LLM systems may incorporate long-term memory mechanisms, such as external databases to store past interactions, allowing the agent to selectively retrieve and use relevant historical context in conversations [26]. While user summaries can improve contextual continuity in conversations, overreliance on user summaries may introduce the risk of information degradation over time. Repeated summarization of prior interactions may lead to loss of nuance, emotional context, or specific behavioral details, which are often critical in behavioral health settings. Therefore, systems incorporating long-term memory should consider strategies to preserve important contextual information, such as periodic reference to current interaction data or structured storage of key variables [26].

In the RENEWAL project, the model’s context window includes the system prompt (defining the health coach’s role and persona), the current session’s conversation history (short-term memory), and retrieval-augmented generation (RAG)–retrieved documents (disease-specific information pulled from a curated knowledge base). This is supplemented by long-term memory in the form of user summaries, which are a running update of the user’s history, health goals, symptoms, and recurring topics. This summary is stored in the database and included in the context window for future sessions.

### LLM Agent and Decision-Making

The user input integrated with system-level prompts and contextual memory is processed by a large neural network (the LLM agent), which uses its transformer architecture to generate contextually relevant text responses [21]. At this stage, the system determines whether additional supporting information is required to generate an appropriate response.

In the RENEW-AI and RENEWAL projects, rather than training a model from scratch, we used a proprietary GPT model available through our university (University of Michigan; UM). While the full architectural background of these models is not yet disclosed, it is known that the GPT family is built upon transformer-based systems described above. To integrate UM-GPT4o into our intervention, we accessed it through application programming interfaces (APIs), which act as a secure data bridge between the mobile app or mHealth intervention and the institutional AI platform. Using a proprietary model via API allowed our study participants to have bidirectional conversations with our LLM-based health coach while substantially reducing the technical complexity, development time, and computational cost for our project. Because the base model’s internal weights were fixed, we focused on safety, clinical relevance, and narrowing the model’s general capabilities to the specific goals of our intervention through refinement of instructional prompts, grounding data, and output guardrails.

### RAG and the Vector Database

RAG is an architectural approach that supplements an LLM’s internal knowledge with an external library or curated knowledge base [29]. This mechanism allows the system to ground its responses in verified intervention materials, thus maintaining clinical accuracy and mitigating the risk of hallucinations (when a model produces factually incorrect information). When the LLM agent receives data input from the user and determines that additional supporting information is needed, it initiates a multistep retrieval process. First, the system converts the user’s text into a numerical representation called an embedding. This embedding is then used to perform a similarity search within a vector database containing the preindexed RENEW program materials and user summaries. The system then retrieves the most relevant “chunks” of text, often referred to as the “top-k” units, and appends them to the prompt. This grounding process increases the likelihood that responses are informed by established clinical protocols and historical user context rather than general probabilistic patterns. However, RAG does not fully eliminate hallucinations, as models may still misinterpret retrieved information or fail to appropriately incorporate provided context. Therefore, RAG can be viewed as a mitigation strategy that improves response reliability but does not replace the need for additional safeguards, such as prompt constraints and output guardrails.

### System Output

Based on the user input and integrated context (prompt, memory, and optional retrieved content), the LLM generates a response or system output. In the RENEW framework, the system output was intended to support reflection, reinforce self-efficacy, and guide behavioral decision-making.

### Output Guardrails

The output guardrails refer to mechanisms designed to constrain the system’s response to ensure safety, clinical appropriateness, and adherence to ethical boundaries. Because LLMs generate responses probabilistically, they are prone to hallucinations, but using guardrails at the levels of data input, system prompts, and output can help reduce the risk of hallucinations.

In the RENEW framework, data inputs are scanned for indicators of self-harm (guardrails at the level of data input). If high-risk language is detected, the system directs the user to appropriate professional resources or emergency services. At the prompt level, safety is maintained through prompts (ie, role-definition and RAG-grounding). Finally, at the output level, system responses are flagged if clinical recommendations fall outside RENEW’s scope (eg, specific dosage recommendations or diagnostic statements). Together, this ensures that the system output remains ethically sound and clinically safe. The system output completes the feedback loop by providing a grounded, supportive coaching response, which influences the user’s behavior and thereby the user’s next interaction with the LLM.

## A Practical Design Workflow for LLM-Based Behavioral Interventions

**Figure 3:**
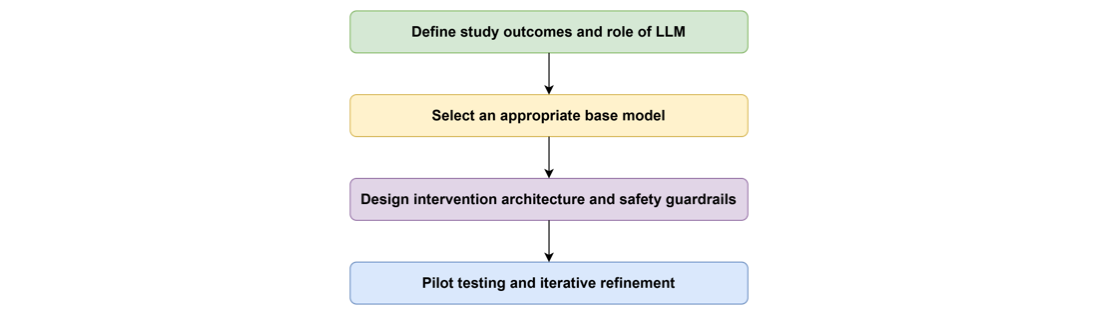
Design workflow for large language model (LLM)–integrated behavioral interventions.

Building on our experience with the RENEW-AI and RENEWAL projects, we propose a 4-phase design workflow to guide the development of LLM-based behavioral interventions (Figure 3).

At the time of writing, our work reflects early implementation efforts, including system design, pilot testing, and initial participant engagement, rather than completed efficacy trials. Accordingly, we present this workflow as a structured approach to early-stage development and responsible integration.

### Define Study Outcomes and the Role of LLM

The first step in integrating an LLM into a behavioral intervention is to clarify its intended behavioral role. Researchers should define the specific function of the model, such as providing reflective coaching, delivering structured education, reinforcing goal setting, or supporting motivational processes. Equally important is explicitly stating what the model is not intended to do, including offering medical diagnoses or treatment recommendations. In parallel, researchers should define the study’s primary and secondary outcomes and articulate how LLM integration is hypothesized to influence those outcomes. These may include direct clinical outcomes, such as, fatigue severity, physical activity, and causal mechanisms (eg, engagement, self-efficacy, or adherence).

### Selection of an Appropriate Base Model

Once the LLM’s role and outcomes are clearly defined, researchers can determine the appropriate technical strategy. This includes selecting the appropriate LLM for the behavioral intervention. As described earlier, our team adapted a proprietary LLM to function as a digital health coach for individuals with SSc [24]. One of the most consequential decisions in this process was selecting the type of LLM to use.

For our project, we began by asking, “Can the needs of our behavioral intervention be fulfilled by the capabilities of existing LLMs?” From there, we reflected on a series of scientific and logistical considerations. Scientifically, we considered issues such as data privacy, control over participant interaction data, the language and tone required for the intervention, the specific role the LLM was expected to play, the extent to which the model would need to be customized, training data needed to refine the model’s response, and finally, implications for transparency and reproducibility of our scientific methods. Logistically, we considered practical constraints including cost and available research funding, development timelines, access to technical expertise, infrastructure requirements, and our capacity to host and maintain an LLM-based intervention over time. Together, these considerations shaped our decision-making process and informed how we ultimately integrated UM-GPT4o into the RENEW platform.

**Figure 4:**
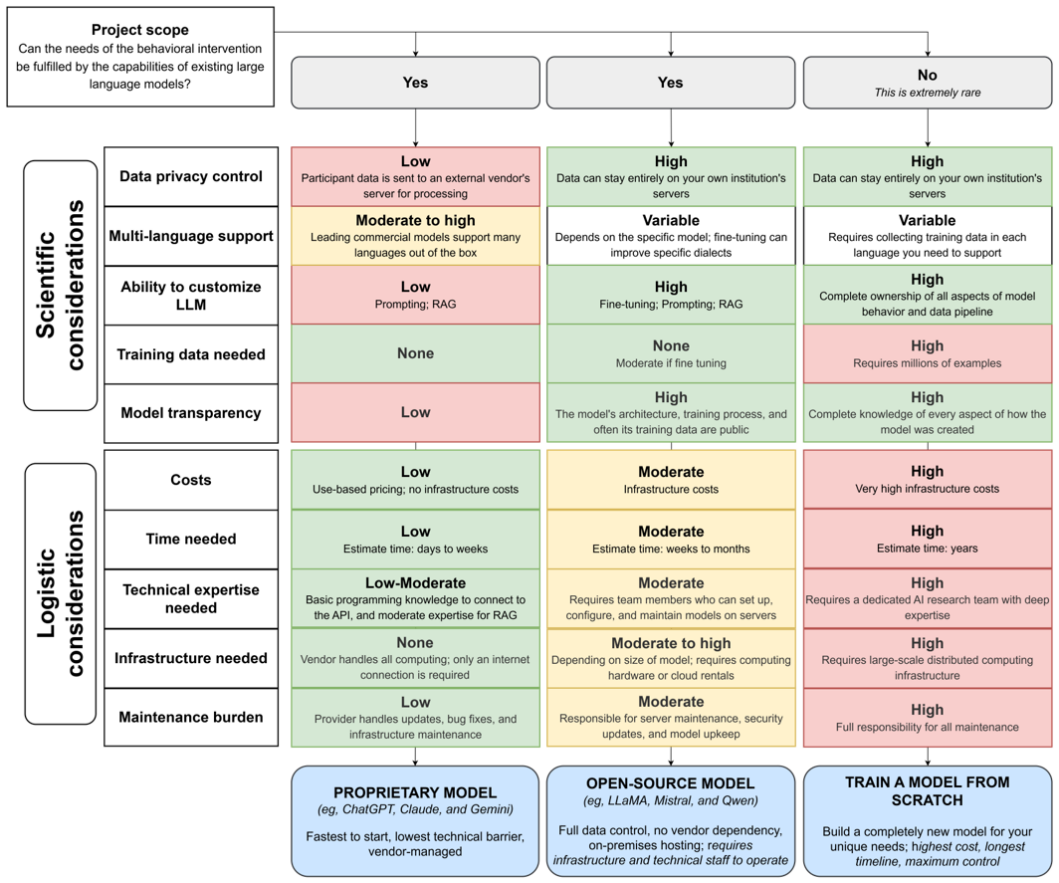
Considerations for deciding what type of large language model (LLM) is most suited for a behavioral intervention. AI: artificial intelligence; API: application programming interface; RAG: retrieval-augmented generation.

As shown in Figure 4, integrating LLMs into behavioral interventions typically involves choosing among 3 approaches: using a proprietary model (eg, GPT-4o and Google Gemini), fine-tuning and hosting an open-source model (eg, Llama and Mistral), or training a foundational model from scratch. The appropriate choice depends on the intervention’s goals, privacy requirements, available expertise, and resource constraints. For many behavioral research teams, adapting an existing proprietary or open-source model is the most practical and scientifically reasonable option. Although training a model from scratch offers the highest degree of control over model behavior and data pipelines, it generally requires substantial data, infrastructure, and specialized expertise. In contrast, hosting and fine-tuning an open-source model allows data to remain on institutional servers and provides greater control over model versioning and updates, which may support reproducibility in research settings. This approach, however, requires institutional infrastructure and technical personnel capable of deploying, maintaining, and securing the system over time. Finally, proprietary models have the lowest technical barrier and minimal infrastructure requirements. They can typically be integrated into existing mHealth platforms relatively quickly and without significant upfront investment in computing resources, which makes them well-suited for pilot studies or early-stage development. However, this convenience may come with reduced transparency regarding model architecture and training data, and less direct control over how updates are managed. Therefore, selecting an appropriate LLM is a methodological decision that balances scientific and logistical considerations within the realities of a given intervention.

### Design Intervention Architecture and Safety Guardrails

After selecting the model, attention shifts to architectural design. This includes developing prompting strategies that define the LLM’s role, tone, and boundaries, as well as implementing guardrails to mitigate risks such as inappropriate medical advice or failure to detect crisis language. Furthermore, decisions related to short-term context windows, long-term memory mechanisms (eg, user summaries), and RAG need to be made. These architectural choices directly influence personalization, safety, and reproducibility.

Regarding safety guardrails, researchers should consider safety risks related to inappropriate or out-of-scope responses and the potential for algorithmic bias arising from training data used in both proprietary and open-source models. Because LLMs are trained on large-scale datasets that may reflect historical and societal biases, model outputs may differentially represent or respond to users based on factors such as language, culture, gender, or health status. In behavioral health contexts, such biases may influence user engagement, perceived empathy, and the relevance of behavioral recommendations, potentially contributing to inequities in intervention delivery and outcomes. Therefore, researchers should consider strategies to identify and mitigate bias, including diverse evaluation samples, human review processes, and ongoing monitoring of model performance across user subgroups.

### Pilot Testing and Iterative Refinement

The final phase involves pilot testing, evaluation, and iterative refinement of the intervention. Depending on the stage of development, researchers may consider feasibility studies, pilot randomized trials, or hybrid effectiveness-implementation designs to assess clinical outcomes and implementation factors, such as adoption, usability, and sustainability. Aligning early system evaluation with these study designs may support the translation of LLM-based interventions into rigorous behavioral and clinical research. A human-in-the-loop review may be important in early implementation to assess response quality, empathic tone, clinical alignment, and safety adherence. Additionally, user-centered design, such as usability testing and iterative feedback from users, may help ensure that these interventions are acceptable, understandable, and responsive to the needs of the target population.

While evaluation metrics for LLM-based behavioral interventions are still evolving, a combination of automated technical assessment with human-centered evaluation is increasingly used in practice [30,31]. For the RENEW intervention, automated evaluation included a structured assessment of response quality to examine relevance, hallucination, and completeness of retrieved data. In parallel, human evaluation included clinician review of responses for fidelity to behavioral strategies (eg, alignment with motivational interviewing principles), as well as assessment of empathic tone, clarity, and clinical appropriateness.

## Conclusions

LLMs offer new opportunities to enhance personalization and adaptability in digital behavioral interventions. However, integrating LLMs into behavioral interventions requires deliberate architectural, methodological, and ethical decision-making. This viewpoint provides a structured synthesis of LLM foundations tailored to behavioral science, translates technical concepts into intervention-relevant design considerations, and grounds these principles in an applied case example. By combining conceptual explanation with practical implementation experience, we aim to offer behavioral researchers a clear and actionable framework for early-stage LLM integration. Along with these strengths, several limitations should be acknowledged. First, the RENEW-AI and RENEWAL projects are in the early stages of implementation, with formal evaluations of effectiveness, safety, and long-term engagement ongoing and therefore not included in this paper. Second, this framework focuses specifically on LLM-based systems and may not generalize to other AI approaches, such as reinforcement learning or unsupervised modeling. Third, although we describe architectural safeguards and grounding mechanisms, ongoing monitoring for model drift, hallucinations, and potential biases across demographic groups is not elaborated on in this paper.

## Data Availability

The manuscript discusses the author’s experiences in integrating artificial intelligence (AI) into a behavioral intervention. No datasets were used in the preparation of this work. There are no associated datasets available for this manuscript.

## Abbreviations

AI: artificial intelligence
API: application programming interface
LLM: large language model
mHealth: mobile health
NLP: natural language processing
RAG: retrieval-augmented generation
RENEW: resilience-building energy management to enhance well-being
RENEWAL: RENEW via Artificial Language
SSc: systemic sclerosis
UM: University of Michigan
